# Changes in Radiology Due to Artificial Intelligence That Can Attract Medical Students to the Specialty

**DOI:** 10.2196/43415

**Published:** 2023-03-20

**Authors:** David Shalom Liu, Kamil Abu-Shaban, Safwan S Halabi, Tessa Sundaram Cook

**Affiliations:** 1 University of Toledo College of Medicine and Life Sciences Toledo, OH United States; 2 Department of Medical Imaging Ann & Robert H Lurie Children's Hospital of Chicago Chicago, IL United States; 3 Department of Radiology Hospital of the University of Pennsylvania Pennsylvania, PA United States

**Keywords:** artificial intelligence, AI, radiology, medical students, residency, medical education, students, automated, clinical informatics, patient, care, innovation, radiologist

## Abstract

The role of artificial intelligence (AI) in radiology has grown exponentially in the recent years. One of the primary worries by medical students is that AI will cause the roles of a radiologist to become automated and thus obsolete. Therefore, there is a greater hesitancy by medical students to choose radiology as a specialty. However, it is in this time of change that the specialty needs new thinkers and leaders. In this succinct viewpoint, 2 medical students involved in AI and 2 radiologists specializing in AI or clinical informatics posit that not only are these fears false, but the field of radiology will be transformed in such a way due to AI that there will be novel reasons to choose radiology. These new factors include greater impact on patient care, new space for innovation, interdisciplinary collaboration, increased patient contact, becoming master diagnosticians, and greater opportunity for global health initiatives, among others. Finally, since medical students view mentorship as a critical resource when deciding their career path, medical educators must also be cognizant of these changes and not give much credence to the prevalent fearmongering. As the field and practice of radiology continue to undergo significant change due to AI, it is urgent and necessary for the conversation to expand from expert to expert to expert to student. Medical students should be encouraged to choose radiology specifically because of the changes brought on by AI rather than being deterred by it.

## Introduction

A 2022 study found that half of medical students who consider specializing in radiology as 1 of their top 3 choices are concerned about the impact of artificial intelligence (AI) on the field [1]. This finding is contrasted by the optimism of leading radiologists from the Association of University Radiologists in 2020 toward AI, citing exciting developments in precision health, workflow efficiency, and decision support [2]. This contradiction highlights a lack of communication between the thought leaders in radiology and medical students regarding the promise of AI in radiology. If medical students only learn about the purported “dangers” and “threats” of AI on the radiology workforce from the media or from nonradiologist physicians, they are at increased risk of believing such false claims, due to their lack of understanding of the complex and irreplaceable roles of the radiologist. The uncertain impact of AI on the future of radiology can further deter medical students from choosing radiology [3,4]. There is an unavoidable need to reconcile this misunderstanding between current physician AI experts and medical students currently in undergraduate medical education who are tasked to choose their specialty.

Yet, it is during this crucial time of transition in the field of radiology that there is an even greater and more urgent need for medical students to rise to the challenge as leaders and innovators. AI is reshaping the practice of radiology, just as picture archiving and communication system and magnetic resonance imaging did in previous decades [5,6]. This reinvention of radiology can create new reasons for medical students to pursue the specialty. However, few articles have highlighted how AI can attract medical students toward radiology rather than scaring them away [7]. Even fewer articles about this topic have been written specifically for medical students, without much technical jargon, as the conversation is mostly *expert to expert* right now [8]. Beyond disproving the myth that AI is going to cause a negative disruption to the future radiology job market, this article encourages current and future medical students to not feel intimidated by AI, but rather be empowered to choose radiology especially because of it. To do so, 2 radiologists who are national leaders on AI and clinical informatics (SH and TC) and 2 medical student leaders (DL and KA) aim to provide a commentary that outlines several novel considerations related to AI that could attract medical students to radiology. Though the focus of this article is solely on radiology, as the influence of AI grows in other specialties, conclusions derived from this article can be applied to other specialties as well.

## The Effect of AI in Radiology

AI is a broad category, encompassing multiple types of technology. The most popular category of AI in radiology is deep learning, which uses sophisticated neural networks to detect patterns in input data and produce outputs [9]. For example, deep learning can learn the relationships between pixels in a chest x-ray to detect findings associated with the presence or absence of pathologies such as cardiomegaly, emphysema, and atelectasis [9,10] at a level similar to practicing radiologists. Though this article will focus mostly on deep learning and pixel-based AI, it is important to note that AI includes natural language processing, which has been successfully applied to report creation, speech recognition, summarization, and text classification in radiology [11].

However, AI for radiology does not exist solely in research but has found its way to clinical utility too, with more than 200 Food and Drug Administration–cleared, commercially available AI software products for radiology available as of early 2023 [12,13]. Nonetheless, a majority of these products still need peer-reviewed publications clearly assessing their clinical utility [12]. However, as radiology research continues to be further polished and clinical use cases developed, it seems inevitable that AI will play a significant role in transforming the field of radiology [9,14]. Recognizing this imminent shift, radiology residencies are now encouraging increased AI literacy in their residents [15,16].

## A Larger Impact on Patient Care

Medical students who want a career during which they can impact the care of the largest number of patients should strongly consider diagnostic radiology, a specialty that is unequivocally integral to the practice of medicine and would potentially benefit from AI. On top of reducing radiation exposure and doses of contrast agents, AI can increase workflow efficiency, improve organ quantification and disease detection, triage exams with urgent findings, and advance precision medicine [12,17]. For example, with an aging population and a greater reliance on imaging in the United States and Canada, there has been significantly increased computed tomography (230%), magnetic resonance imaging (304%), and ultrasound (164%) imaging use within the last 2 decades [18]. The World Health Organization estimates that the percentage of the world’s population that will be over 60 years old in 2050 will be nearly double what it was in 2015 [19]. To match the greater demand, AI can significantly improve the efficiency of the workflow of radiologists. For example, AI for bone age estimation resulted in up to 40% reduction in reading time [20]. Another AI to detect pulmonary metastases reduced reading time by 21% [21]. Furthermore, the World Health Organization data suggest that in 2050, two-thirds of the world’s population over 60 will be in lower- and middle-income countries, increasing imaging volumes in these areas without a proportionate increase in radiologists and radiology trainees [19,22]. For students passionate about global health, RAD-AID is a global health initiative that provides AI tools and associated education to health care providers in lower- and middle-income countries [22]. For countries such as Guyana, which has 750,000 citizens but no in-country radiology programs, RAD-AID helped by starting residency programs and concurrently introducing AI education to help with the significant need to interpret high numbers of imaging examinations [22]. Finally, faster image acquisition by removing image noise [23] and improving image reconstruction [24], combined with decreasing necessary radiation exposure and contrast dose [25], can improve the imaging experience and decrease the risk of side effects for patients. Reducing barriers to imaging increases the value of radiology to both clinicians and patients and can accelerate the pace of diagnosis and treatment. The ability to play a role in the health care of a larger number of patients can give future radiologists a greater sense of meaning and a positive impact.

## A New Need for Innovators and Researchers

AI has created a new space for innovators within radiology. When electronic medical records were introduced in the early 2000s, physicians were neither sufficiently consulted nor involved in the process and did not effectively or actively advocate for themselves and their patients [14]. The promise of data sharing and workflow improvements was not realized, and instead clinicians found themselves working harder—and for the computer, rather than the other way around—to deliver optimal care and create a healthy patient-physician relationship. Without the input of radiologists and physicians from other imaging specialties, such as cardiology, pathology, dermatology, and ophthalmology, the same could happen with AI. Instead, radiologists must lead AI innovations in their specialty [14] and guide data scientists and AI developers in building solutions that directly impact care delivery and improve patient outcomes.

There has been an explosion of radiology AI research in the past few years. Participation in AI research is not limited to radiologists with formal training in AI, computer science, or software development. In fact, the opposite is true; one criticism of radiology AI development has been that anyone with a graphics processing unit and some data can build a working model. While this is technically true, building imaging AI models that make logical sense in the clinical workflow and address an unmet, relevant clinical need requires a domain knowledge that radiologists must provide. Imaging AI is a highly interdisciplinary field, consisting of experts from engineering, computer science, medicine, and informatics. The camaraderie and sharing of knowledge between these experts should be the norm in radiology AI research. For example, at Stanford’s Center for Artificial Intelligence in Medicine & Imaging, leaders in medicine, education, business, computer science, ethics, and linguistics converge to form interdisciplinary teams dedicated to teaching AI and conducting AI research to solve health care issues [26-28]. This sharing of knowledge between fields of expertise creates ever-expanding areas of personal growth and discovery for the physician. Finally, for students who have an entrepreneurial spirit, the number of AI startups in radiology has significantly increased since the AI “boom” in the late 2010s. The 2021 global market size for AI in medical imaging is $1.06 billion, with an expected compound annual growth rate of 46%, leading to a market size of $10.14 billion by 2027 [29]. AI creates the opportunity for new exploration and innovation in various frontiers in medicine, and radiology stands to gain the most from these new developments. For medical students who desire to be creative, lead innovation, and conduct interdisciplinary research, radiology is a field that is filled with such new opportunities.

## The Patient-Facing Radiologist

Radiology is also a highly technical field. Medical students perceive radiologists to have “little or no patient interaction” and think that radiology “is best suited for introverted people” [30]. AI can automate repetitive tasks currently performed by radiologists, such as screenings and lesion or organ measurements [9,14,31]. The hope is that this automation will create time for radiologists to meet and speak to patients face-to-face, to discuss the need for imaging, and to review the results and consult on the next steps in care. In total, 84% of surveyed patients have reported interest in meeting with a radiologist to discuss their imaging findings, with 57% of those willing to pay extra [32]. In 2022, the European Society of Radiology released a statement to encourage further radiologist-patient communication, in light of new technologies such as AI [33]. Although breast imaging and interventional radiology already afford these subspecialist radiologists face time with patients, AI may increase the likelihood that other subspecialties are able to interact with patients because of the time reduced from purely diagnostic work in front of a screen [24,34,35]. No longer just the “doctor’s doctor,” radiologists will be able to demonstrate their value as the patient’s physician via direct patient interaction [35]. Patients will have the opportunity to learn about their diagnosis from the radiologist’s point of view. Furthermore, with the passing of the 21st Century Cures Act, patients now have immediate access to their test results, including their radiology reports. This provides even more needs and opportunities for patients to connect with radiologists. Medical students who are concerned about the relatively secluded nature of the specialty should be encouraged by the potential positive impact AI can have on increasing the quality and amount of patient interactions.

## Becoming Master Diagnosticians and Information Experts

The practice of radiology requires the analysis of multiple sources of data and the integration of the presented information to identify a likely diagnosis. Rather, beyond patient history and the radiologist’s clinical experience, output from AI will be a new source of data that adds to the richness of information presented to the radiologist as they exercise their clinical judgment. AI can also make simple but impactful changes, such as augmenting hanging protocols, which dictate how current and prior examinations (and now, AI results) are displayed to a radiologist for interpretation [17]. Medical students who enjoy becoming the epitome of an information specialist will find radiology augmented by AI to be one of the most intellectually stimulating fields in medicine. They will be expected to consolidate multiple additional sources of quantitative data in addition to reviewing the original reconstructed pixel data. Additionally, they will have the opportunity to translate this into actionable recommendations for both referring clinicians and patients. For example, an electronic health record–based AI can notify the radiologist about the patient’s multiple pulmonary embolism history [36]. With this information, the radiologist will have a higher suspicion in diagnosing pulmonary embolism. Gathering these various sources of information and recognizing the potential for an underlying malignancy, the resident could report that concern so that the patient undergoes a more thorough checkup for potential malignancy. Already integral to the health care system, radiologists will become even more essential, which could improve the work satisfaction and sense of fulfillment of future radiologists.

## For Medical Educators

Medical educators guide medical students as they decide on which specialty to pursue. Mentorship has been shown to be beneficial in a medical student’s medical school experience and a significant factor for career path development for the past few decades [37]. Medical educators must have a holistic understanding of the potential effect of AI on certain specialties in order to disseminate unbiased information and properly advise medical students. Due to the COVID-19 pandemic, web-based learning has become a mainstay, and education on various topics is now much more readily available. For example, a working understanding of AI and its impact on medicine can be gained from free massive open online courses [8]. Additionally, many articles have been recently published to introduce the concept and promises of AI at a level for medical professionals with minimal prior understanding of AI [14,38,39]. To better guide medical students, it would be wise for medical educators to be cognizant of changes in the medical field by staying in touch, by reading and keeping up to date with these new review articles. However, the bottom line is that medical educators should lead by example for medical students by approaching the topic of AI in medicine with a healthy optimism and with critical thinking, given the significant role they can play in informing the future of their medical students.

## Conclusion

Radiologists who embrace AI are unlikely to be replaced by the technology. Instead, AI is poised to positively transform the field. This will create a greater demand for radiologists who are innovators, multidisciplinary researchers, and empathic physicians who take pride in being information specialists. Medical students considering what specialty to choose should be aware of these changes, especially as misinformation spreads about the future impact of AI on the field of radiology. Medical educators should also be cognizant of these changes to properly mentor medical students in deciding their future specialty. Though the current medical student community seems hesitant in pursuing radiology due to AI [1], the positive changes to the field of radiology due to AI can actually create new reasons that may attract medical students toward this specialty. Radiology is currently undergoing a process of transformation. Thus, it is in this crucial time that correct guidance has to be given to encourage current medical students to choose radiology and become both leaders and advocates for positive change.

## Abbreviations

AI: artificial intelligence
